# Primary sclerosing encapsulating peritonitis: a case report 

**DOI:** 10.1186/s13256-023-04020-x

**Published:** 2023-07-14

**Authors:** T. Pintar, M. Tavčar, A. Šušteršič, M. Volavšek

**Affiliations:** 1 Medical Faculty, University of Ljubljana, Ljubljana, Slovenia; 2 University Medical Center Ljubljana, Ljubljana, Slovenia

**Keywords:** Abdominal pain, Intestinal obstruction, Peritoneal fibrosis, Sclerosing peritonitis, Treatment

## Abstract

**Background:**

Sclerosing encapsulating peritonitis is a rare condition with a typical macroscopic appearance, with fibrocollagenous membrane enclosing loops of the small intestine, causing intestinal obstruction. Unexplained recurrent abdominal pain, obstruction, and a large array of other possible clinical signs and symptoms make sclerosing encapsulating peritonitis a diagnostic challenge.

**Case presentation:**

A 48-year-old man of Persian ethnicity was admitted multiple times to the emergency surgery department due to recurrent sudden abdominal pain and chronic obstruction without significant findings in medical history or clinical evaluation. Computed tomography was positive for proximal jejunal dilatation and duodenojejunal flexure stenosis due to internal mesenteric hernia. Exploratory laparoscopy, followed by laparotomy, confirmed thick membrane-like fibrous tissue with complete small intestinal loop envelopment. Extensive membrane excision and adhesiolysis was performed, but no mesenteric herniation was found. Early postoperative paralytic ileus with introduction of low-dose steroid therapy, based on histopathological and immunological results, confirming type III sclerosing encapsulating peritonitis, was completely resolved.

**Conclusion:**

Sclerosing encapsulating peritonitis is a rare and difficult-to-diagnose condition, further divided into primary and secondary sclerosing encapsulating peritonitis, on the basis of underlying etiology, dictating treatment modality and prognosis. Intraoperative diagnosis and surgical treatment are mandatory, besides a wide variety of abdominal computed tomography scans, inconclusive results, and clinical presentations. There are so far no known specific markers for the diagnosis of sclerosing encapsulating peritonitis.

## Background

Primary sclerosing peritonitis (PSP) is a rare clinical entity, with nonspecific radiological markers for which we currently have no noninvasive clinical markers. Early recognition and recurrence in mild form allow medical treatment. Extensive surgical resections in the absence of recognition of the clinical entity result in significant morbidity with consequent short bowel syndrome and dependence on total parenteral nutrition.

### Introduction

Sclerosing encapsulating peritonitis (SEP) is a rare condition in which chronic inflammation causes fibrocollagenous membrane formation around the small intestine, leading to small intestine entrapment and therefore gastrointestinal obstruction at different levels with resulting clinical presentations [1–3]. Large variation in symptoms and clinical signs [1, 4] and spontaneous clinical improvement combined with disease relapses pose a difficult diagnostic challenge. According to the risk factors for the disease, there is a clinical division into primary (idiopathic) and secondary SEP; secondary SEP had a similar clinical presentation to primary one and is related to high-risk conditions, most frequently to the peritoneal dialysis (PD)-related condition abdominal tuberculosis. Further candidate risk factors for primary PSP include extensive endometriosis with many complications, including major spontaneous cyclic and extra-cyclic bleeding, multiple abdominal surgical procedures, and intraabdominal infections. Among the disease-defining factors in the occurrence of idiopathic PSP in association with endometriosis, the role of prostaglandin (PG) E2 receptor and protease-activated receptor (PAR) remains unexplored. In addition, under hypoxic conditions, extensive fibrosis might be directly related to the prostaglandin (PG) E2 receptor and protease-activated receptor (PAR) activity and to epithelial–mesenchymal transition (EMT), thrombin, and PAR1 agonist. These factors have been linked to inflammatory mediators in retrograde menstrual fluid. There is a proven link that hypoxic and proinflammatory stimuli induce epithelial–mesenchymal transition, cell migration, and inflammation as well as subsequent membrane formation resulting in abdominal cocoon formation. Studies have confirmed that circulating miRNAs could be interfered with to demonstrate increased mesenchymal activity in endometriosis, which could also be indirectly linked to peritoneal sclerosis activity.

Candidate factors associated with the development of primary PSP include infectious factors, the use of certain medications, and autoimmune factors or responses. Besides, the proposed etiopathogenesis of SEP includes also developmental disorders related to vascular anomalies and omental hypoplasia. Among factors related to higher incidence of the disease are the use of beta-blockers, abdominal trauma/surgery, ventriculoperitoneal shunt, peritoneal tuberculosis, sarcoidosis, systemic lupus erythematosus, gastrointestinal malignancy, and liver cirrhosis with different etiopathologic triggering factors and pathophysiological mechanisms.

Up to now, the diagnosis is mostly made during surgical exploration; various radiological diagnostic methods are unreliable for diagnosis, including an abdominal computed tomography (CT) scan that, among others, showed better specificity and sensitivity and might play an important role preoperatively [4] in case of clinical suspicion. Also, imaging signs, including thickening and calcification of the peritoneum and dilation of bowel loops with thickening and calcification of bowel walls, are not specific and are associated with other, otherwise clinically more common clinical conditions or diseases, further complicating early and effective diagnosis and appropriate therapeutic interventions.

We report the case of a 47-year-old man of Persian ethnicity who presented to the emergency department several times, complaining of abdominal pain and long-ongoing constipation. Radiologic imaging suggested an internal herniation at the level of the duodenojejunal flexure, but it was not present at the time of explorative laparotomy due to complete upper intestinal obstruction. The diagnosis was made intraoperatively, confirmed promptly by histopathology. Extensive adhesiolysis with careful membrane excision and a short course of postoperative steroid therapy was efficient in postoperative paresis resolution and functional recovery of the small intestine. Treatment was based on histopathological evaluation.

## Case description

A 48-year-old man of Persian ethnicity presented multiple times to an outpatient surgical emergency department (ED) with complaints of colicky abdominal pain in the left upper and lower quadrant, which became worse after a meal, nausea, vomiting, and a history of obstipation, which lasted 3 months before his first episode of abdominal pain. The patient had no previous history of abdominal pain or abdominal surgery. He also had no significant medical history of chronic diseases such as hypertension, obesity, or diabetes, only occasional problems with acid reflux disease. The patient denied using any medications, drugs, or alcohol, or having any allergies. The patient was examined at the emergency department (ED) four times with very similar complaints and diagnostic findings. There were no alarming findings on physical examination, only abdominal tenderness and pain in the left upper and lower quadrants. Bowel sounds were normal in all four quadrants.

Laboratory blood findings were insignificant. An abdominal X-ray suggested obstipation, but there were no signs of intestinal obstruction. The patient was treated with an osmotic laxative, a proton pump inhibitor (PPI), an analgesic, and a spasmolytic. After that, he felt relief and was discharged home with instructions in case of further abdominal pain, fever, or vomiting. He was also referred for further diagnostic investigation.

**Fig. 1:**
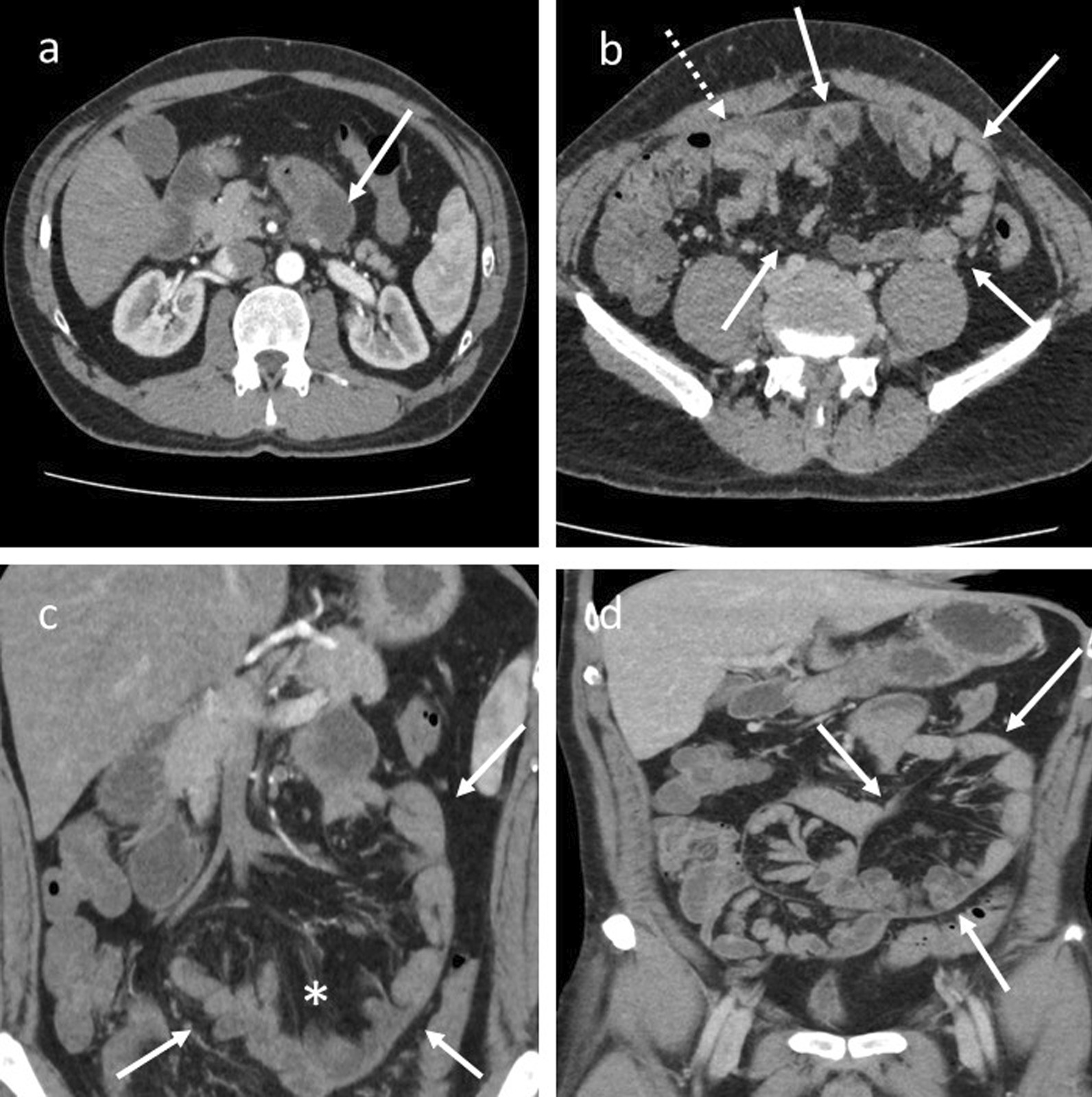
Contrast-enhanced computed tomography (**a**–**d**). **a** Axial cross-section demonstrating dilated proximal bowel loops (arrow). **b**–**d** Axial cross-section and coronal-cross section demonstrating “sac-like” configuration of small bowel loops (arrows), free fluid (dashed arrow), and mesenteric fat stranding (asterisk)

The patient underwent gastroscopy, colonoscopy, abdominal ultrasound, and abdominal computed tomography (CT). Gastroscopy showed erythematous gastric mucosa and fluid retention in small bowel loops. Colonoscopy revealed no pathological findings. Abdominal ultrasound suggested subileus of the small bowel, which is why the patient underwent further abdominal CT. An abdominal CT scan was suggestive of duodenojejunal flexure stenosis due to an internal mesenteric hernia (Fig. 1a–d). Proximal small bowel loops were dilated at 3.5 cm; distal loops were of normal diameter, with “sac-like” configuration, and contained mesenteric fat stranding. There was a small amount of free fluid and no visible free air. No enlarged mesenteric lymph nodes were found. Because of the patient’s clinical and correlated radiological findings, he was prepared for surgery and later underwent exploratory laparoscopy.

During exploratory laparoscopy, we found thick membrane-like fibrous tissue, enveloping loops of the small intestine and adhesions between the loops of the small intestine. Due to the nonrecognition and inaccessibility of structures in the abdominal cavity, covered with a thick fibrous membrane, we decided to proceed with the conversion to laparotomy and performed vast membrane excision and adhesiolysis. The fibrous membrane was removed, and adhesions between small bowel loops were released. We also took tissue samples and peritoneal swabs for histopathological and microbiological examination. Although the CT scan suggested internal mesenteric hernia, we did not find any during the operation despite careful and thorough observation. We also did not find any congenital small intestinal pathologies.

**Fig. 2:**
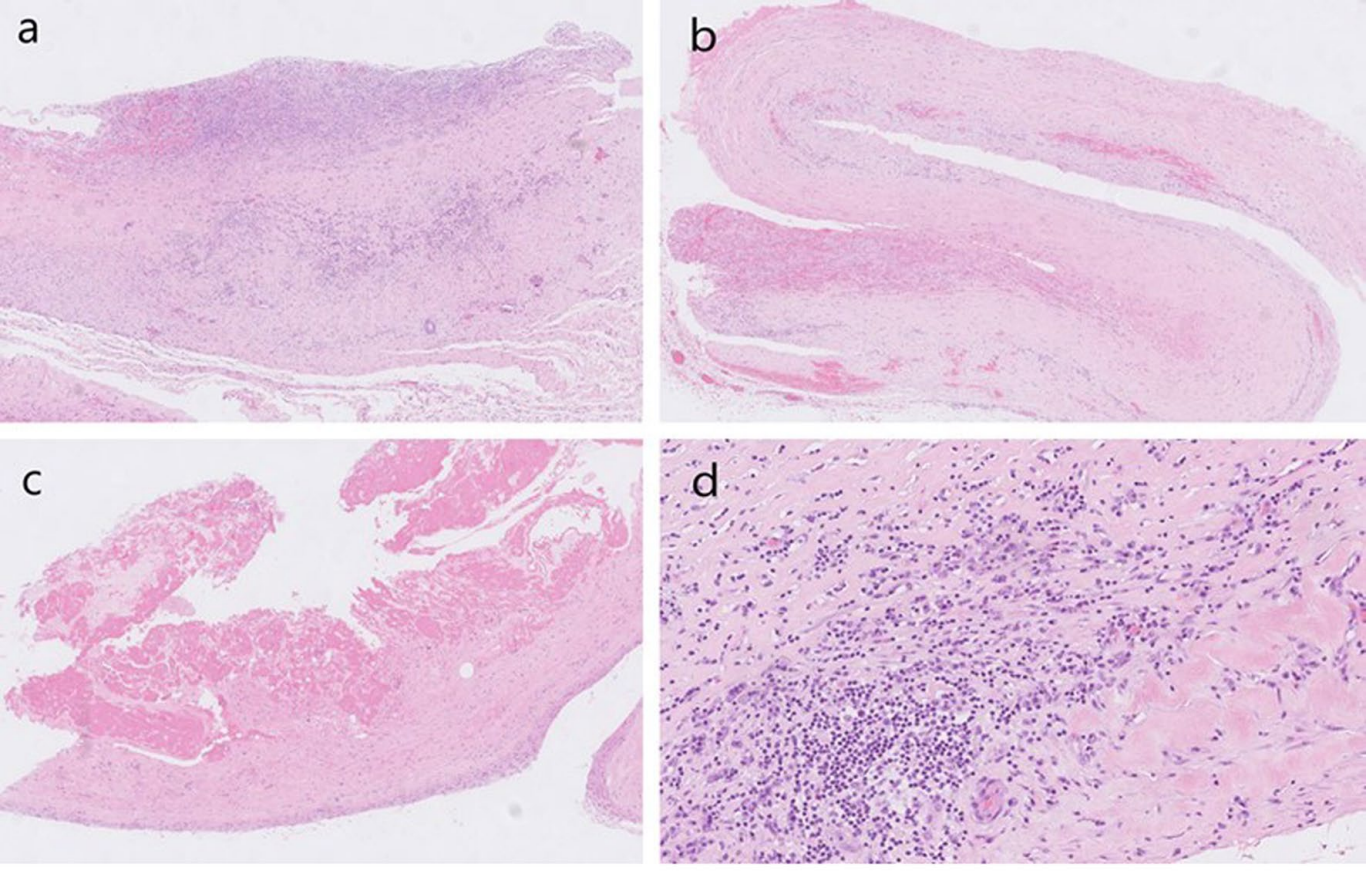
Variable morphology of chronic fibrinous sclerosing peritonitis (**a**–**d**). **a** Low-power view of chronic fibroproductive peritonitis showing peritoneum focally covered with fibrin, adjacent granulation tissue, and mononuclear inflammatory infiltrate, Original magnification, ×4. **b** A predominantly sclerosed segment of resected peritoneum with mild fibrin exudate, Original magnification, ×4. **c** Thickened fibrotic peritoneum covered with fibrin, practically without inflammatory cells, Original magnification, ×4. **d** Sclerotic background with focal lymphoplasmacytic inflammatory infiltrate mixed with eosinophils suggestive of IgG4 related disease, not confirmed after further investigations. Original magnification, ×20

The patient was treated with complete parenteral nutrition for an early postoperative course of 10 days, followed by a combined parenteral and liquid oral diet until complete recovery. A short course of prolonged antibiotic surgical prophylaxis with cefazolin and metronidazole was followed by an amoxicillin course for 7 days, based on intraabdominal intraoperative swab culture results that were positive for aerobic *Escherichia coli*, *Klebsiella oxytoca*, and *Enterococcus faecalis* and negative for anaerobic culturing. In a short, early postoperative course, the patient complained of persistent nausea. Abdominal ultrasound indicated dilated small bowel loops and no peristalsis. An abdominal X-ray on day 9 postoperatively suggested paralytic ileus due to recurrent duodenojejunal flexure stenosis. Peripheral venous blood showed normal SIgA (serum immunoglobulin A) 2.95 g/l (range 0.61–3.56 g/l), elevated S-IgG4 3.1010 g/l (range 0.30–2.1010 g/l) and elevated Il-6 97.9 ng/l (range up to 7.0 ng/l). (ELISA), a serum iron level of 2.1 micromol/l (range 10.7–28.6 micromol/l) and a normal haematological profile (CRP 5 mg/l, range 5 mg/l). Taking into account the histopathological findings, the levels of S-IgA, SIgG-4 and Il-G (interleukin-6) in the peripheral venous blood and the clinical picture, steroid treatment of PSP was initiated at a dose of 1.5 mg per kg body weight (bw) with dose tapering depending on the resolution of the paralytic bowel obstruction 2 weeks after surgery. The patient gradually obtained regular defecation habits and oral nutrition and reported no nausea, so we discharged him on day 22 postoperatively. Peritoneal swaps were tested for acid-fast bacteria. The results were negative. Cultivation of mycobacteria from peritoneal fluid was also made and tested negative. Peritoneal tissue samples were stained with hematoxylin and eosin. Histologically, chronic fibrinous sclerosing peritonitis with an uneven and relatively mild lymphoplasmacytic inflammatory infiltrate was found (Fig. 2a–d). Kongo staining for amyloid was negative, as was immunohistochemistry for amyloid A, transthyretin, and kappa and lambda light chains. Plasma cells were polyclonal. Staining for IgG and IgG4 with subsequent counting of positive cells on four high-power fields (HPFs) revealed approximately 17 IgG4 plasma cells per HPF, with an IgG4/IgG ratio of 39%. A histological report suggested the diagnosis of type III sclerosing encapsulating peritonitis or “abdominal cocoon syndrome,” which was etiologically unexplained (idiopathic).

Other differential diagnostic possibilities such as amyloidosis and other forms of immune-borne diseases, subclinical primary virus peritonitis, or complication from beta-blocker treatment were excluded.

The patient received instructions on medication in case of recurrence of the same symptoms at the time of hospital discharge. At outpatient follow-ups, months 1, 3, and 6 postoperation, the patient reported no pain, normal bowel movements, and a return to normal weight before the onset of the disease. Laboratory results showed no abnormalities in haemogram, electrolytes, hepatogram. Exceptionally, he returned to the outpatient clinic again 10 months after hospital discharge for anal thrombosis unrelated to the underlying disease.

## Discussion

SEP, also referred to as abdominal cocoon, abdominal cocoon syndrome, peritonitis chronica fibrosa incapsulata, sclerosing peritonitis, or encapsulating peritoneal sclerosis, is a chronic inflammatory condition, characterized by a fibrocollagenous membrane that forms around loops of the small and/or large intestine, causing abdominal pain and intestinal obstruction on different levels [1, 3, 5]. SEP is further divided into primary (idiopathic) and secondary SEP [1]. Primary SEP is not linked to any known causes; the only known role by far is that of cytokines and fibroblasts in fibrocollagenous tissue formation and neoangiogenesis [1, 2]. The term abdominal cocoon or abdominal cocoon syndrome refers to primary SEP [1]. Secondary SEP is more common and is closely correlated with any abdominal or systemic factors that cause chronic inflammation in the peritoneum or its surroundings [1]. Factors strongly correlated with SEP are previous abdominal trauma or surgery, abdominal tuberculosis or sarcoidosis, chronic beta-blocker usage, cirrhosis, liver transplantation, abdominal malignancy, various autoimmune disorders, and more [1, 2, 6, 7]. However, the most commonly observed and reported correlation is definitely with long-term peritoneal dialysis [8]. SEP is a very rare condition, which contributes to a difficult and late diagnosis [1]. The previous literature review of SEP lists only 206 case reports or reviews from 1980 to 2020; the suggested incidence is up to 3%, and mortality in patients on peritoneal dialysis is up to 51% [3]. Most cases in Slovenia are reportedly linked to long-term peritoneal dialysis [9]. Recent case reports also suggest a correlation between SEP and recovery after severe coronavirus disease 2019 (COVID-19), as severe acute respiratory syndrome coronavirus 2 (SARS-CoV-2) is associated not only with the respiratory system but also with the gastrointestinal system [10]. Authors report cases with primary SEP after recovery from severe COVID-19 pneumonia and no other comorbidities, backed up by a positive reverse-transcription polymerase chain reaction (RT-PCR) result on peritoneal fluid analysis [10]. SEP is, to date, a very difficult diagnosis to make preoperatively, largely due to the vast variety of reported symptoms and clinical signs, and is usually an unexpected finding during surgical exploration due to signs of intestinal obstruction [1, 11]. Some patients can be asymptomatic for a long time, but the most usual presentation is recurring abdominal pain and acute, subacute, or chronic obstruction without any other concerning abnormalities in the patient’s history or clinical findings [1, 11]. Patients can also present with anorexia, nausea, vomiting, or ascites [1, 11]. SEP can sometimes also present as acute abdomen syndrome [1, 11, 12].

Some case reports also present cases of patients with SEP and only a few or none of the aforementioned symptoms and clinical signs, which confirms a wide array of different clinical presentations of SEP [5, 13]. Our patient presented with recurrent abdominal pain and chronic obstruction, but his problems were successfully managed with conservative treatment. He then underwent further diagnostic investigation to identify an underlying cause for repeating problems.

SEP is usually diagnosed only during exploratory laparoscopy [3, 14]. Laboratory findings are nonspecific but may include electrolyte imbalance or increased creatinine as a result of dehydration and anorexia [14]. X-ray findings are also nonspecific [15]. Contrast-enhanced abdominal CT presents the most useful tool for preoperative diagnosis of SEP [15, 16]. The most common findings are peritoneal thickening, enhancement, and calcification [15–18]. Peritoneal thickening and fibrosis lead to small bowel loop entrapment and congregation; adhesions can form, and the small bowel is then placed in the midline [1, 15, 16, 18]. This finding is called the “cocoon sign” or “cauliflower sign” [14, 16, 18]. Another possible, although not so common, sign is the “bottle gourd sign,” in which duodenal dilatation is seen if the obstruction forms proximally in the jejunum [19]. Magnetic resonance imaging (MRI) findings are very similar to those in CT imaging [15]. Our CT findings were very insignificant. They suggested proximal small bowel loop dilatation and duodenojejunal flexure stenosis due to mesenteric herniation. Mesenteric herniation is an important differential diagnosis in abdominal imaging of SEP [15, 17, 20]. No mesenteric herniation was found in our case during a thorough exploratory laparoscopy or following laparotomy. The correct diagnosis in most cases is still intraoperative and histopathologic [1, 2]. Intraoperative findings usually include a thickened peritoneum with a “leathery” appearance and encapsulated small bowel loops, possibly with adhesions, while histopathologic findings show a predominance of fibrotic tissue with little or no inflammatory cell infiltration [1, 15]. Our intraoperative and histopathologic findings were matching, and the diagnosis of primary SEP was confirmed after further exclusion of possible differential diagnoses.

Currently, we do not have any specific markers that might be used for the diagnosis of SEP [1, 3]. According to the literature, there are different possible mechanisms involved in SEP, among them inflammation dependent [Jennings *et al*.] and immunologically mediated [Manzano *et al*.]. Based on the literature search, there are newer descriptions on the possible role of immune-mediated SEP with the basic concept of autoimmune mechanisms involved; in our case presentation, we also support autoimmune concepts of disease as we confirmed histopathologically approximately 17 IgG4 plasma cells per HPF with an IgG4/IgG ratio of 39% [22–25]. We support the immune mechanisms involved in SEP, excluding other optional inflammatory mechanisms involved in SEP pathophysiology, as described by Jennings *et al*. [21]. Besides, based on the results of the cultivation of abdominal free liquid, positive for the aforementioned bacteria, the isolated culture might be the consequence of bacterial translocation due to prolonged inflammation, intestinal stasis, weight reduction, and recurrent antibiotic treatment for clinical signs of abdominal pain, vomiting, and fever with a negative radiological evaluation. Finally, we also support the autoimmune mechanism involved in SEP with efficient treatment, that is, disease course resolution after steroid introduction. Nine months after treatment, no recurrence of clinical signs has been reported. We are limited in our evidence.

We are limited in presentation as we did not analyze plasma IgG4 concentration, even though, according to the literature, it is a useful tool supporting SEP diagnosis after exclusion of other possible diseases [22].

New diagnostic tools are mandatory to reduce long-term complications, both medical and surgical, and early medical treatment is mandatory [26]. The current literature search does not offer any algorithms for the early diagnosis and medical treatment of SEP [3].

Treatment and management of SEP are based on the underlying etiology, severity of the case, and the patient’s clinical signs and symptoms [1, 27]. There is still no firm agreement on the treatment of primary SEP [1, 27, 28]. Asymptomatic patients, patients with mild symptoms, patients in the early stages of the disease, and those with negative results of imaging modalities or poor surgical candidates can be successfully treated conservatively [27, 28]. The conservative treatment consists of parenteral nutritional support, steroid therapy, tamoxifen, and anti-inflammatory drugs such as azathioprine, colchicine, or mycophenolate mofetil [1, 3, 28, 29]. Based on the laboratory data, histopathology data, and results of abdominal CT, including IgG4 levels of 3.101 g/l that represent a 1.5× increase in peripheral venous blood and an IgG4/IgG ratio of 39% in histopathology, at the same time as Il-6 levels of 97.9 ng/l, representing a 14× increase, we can conclude that SEP in the presented case might not be included in the pool of IgG4-related diseases but rather represent hyper-IL-6 syndrome with an abundant inflammatory response in the peritoneal membrane, as well as inflammatory infiltrate mixed with eosinophils and abundant fibrin. The introduction of steroid treatment resulted in prompt reductions of Il-6 and IgG-4 in peripheral venous blood and clinical manifestations. Postoperative follow-up values were within the normal range. Tsukuda *et al*., as well as data and literature, including case reports and a literature review, support our conclusions from the described case presentation study that, in SEP, the implication of the investigative combination IL-6 and IgG4 might be used as an activity marker and predictor of the disease [30, 31]. Also, a reduced value of serum iron in the presence or absence of anemia speaks in favor of the prevalence of chronic inflammatory events in the presence of a normal CRP value. Conclusions per se offer earlier diagnostic and nonsurgical treatment modalities, as described in the literature. Finally, from the aspect of IL-6 diseases, lymphoproliferative diseases should be excluded [30, 32].

Surgical intervention remains the gold standard for management of primary SEP [27, 28]. Most patients undergo exploratory laparoscopy after unexplained recurrent episodes of intestinal obstruction [15]. The most suitable procedure in the following laparotomy is extensive fibrocollagenous membrane excision and adhesiolysis [27–29, 32]. Small bowel resection is generally not advised but should be considered in cases of necrosis development [27, 32]. Our patient underwent a necessary exploratory laparoscopy due to complete small intestinal obstruction and clinical deterioration and to find an underlying cause of recurrent abdominal pain and chronic intestinal obstruction, followed by laparotomy with extensive membrane excision and adhesiolysis. The patient later developed early postoperative small bowel obstruction (EPSBO), which presented as persistent nausea and was confirmed using ultrasound and X-ray. EPSBO is a possible early postoperative complication after vast membrane excision and adhesiolysis, and our patient was, as advised in current literature, successfully treated with complete parenteral nutrition and low-dose steroid therapy [1, 3, 5, 29–32].

Abdominal CT has high specificity and should be useful as a tool in preoperative diagnostic modalities. So far, no serum markers have been described to be used as diagnostic criteria; our case report study showed increased IL-6 and IgG4, and low serum iron combined with a normal CRP level, which might be an important diagnostic criterion and support other newer descriptions in the literature; IL-6 diseases should be excluded before the introduction of conservative treatment. To date, most SEP cases are still diagnosed during exploratory laparoscopy, and for advanced disease, surgical membrane excision and adhesiolysis remain the gold standard. The high burden of disease in women with proven endometriosis and complications of the disease, in addition to the clinical picture described above, requires consideration of evidence from the literature and the collection of candidate markers that could be used to infer the occurrence and prevalence of the disease, taking into account the triggering factors associated with fibrous tissue overgrowth in the abdominal cavity.

## Conclusions

SEP is a rare but very important differential diagnosis in patients presenting with unexplained recurrent abdominal pain, vomiting, and intestinal obstruction on different levels. The collection of candidate markers that could be used to infer the occurrence and prevalence of the disease is needed, taking into account the triggering factors associated with fibrous tissue overgrowth in the abdominal cavity.

Finally, we encourage all experts to add primary SEP to their differential diagnostic pool in patients with unexplained abdominal pain and obstruction and use the optional diagnostic tool before starting early conservative treatment.

## Data Availability

Not applicable.

## Abbreviations

SEP: Sclerosing encapsulating peritonitis
CT: Computed tomography
ED: Emergency department
PPI: Proton pump inhibitor
BW: Body weight
RT-PCR: Reverse-transcription polymerase chain reaction
MRI: Magnetic resonance imaging
EPSBO: Early postoperative small bowel obstruction
